# Case report: Coronary allograft vasculopathy: an accurate reflection of the histopathological findings on cardiovascular magnetic resonance imaging

**DOI:** 10.3389/fcvm.2023.1123212

**Published:** 2023-05-17

**Authors:** Carmela Napolitano, Giorgia Grutter, Paola Francalanci, Antonio Amodeo, Aurelio Secinaro

**Affiliations:** ^1^ Advanced Cardiovascular Imaging Unit, Bambino Gesù Children’s Hospital, IRCCS, Rome, Italy; ^2^ Heart Failure, Transplantation and Cardio-Respiratory Mechanical Assistance Unit, Bambino Gesù Children’s Hospital, IRCCS, Rome, Italy; ^3^ Pathological Anatomy Unit, Bambino Gesù Children’s Hospital, IRCCS, Rome, Italy

**Keywords:** transplantation, heart, cardiac magnetic resonanance, chronic rejection, specimens anatomical

## Abstract

Heart transplant recipients undergo extensive invasive and non-invasive postoperative screening to exclude complications, such as allograft rejection and vasculopathy. Cardiac magnetic resonance imaging is a non-invasive, non-irradiating, diagnostic tool for monitoring graft health and identifying possible tissue rejection or myocardial fibrosis. We describe the case of a 29-year-old female heart transplant recipient admitted to our care center with a worsening clinical condition. The patient underwent clinical evaluation, blood tests, including troponin I and N-terminal pro brain type natriuretic peptide, transthoracic echocardiography, invasive coronary angiography, and cardiovascular magnetic resonance imaging. Cardiovascular magnetic resonance imaging showed widespread sub-epicardial hyperintensity of the myocardial segments along the course of the coronary arteries. T2 mapping sequences showed an elevated value and the myocardial native T1 values and extracellular volume percentage were significantly increased. Late gadolinium enhancement demonstrated a diffuse sub-epicardial hypersignal along the lateral, free, and left ventricular walls. All the sequences evidenced widespread hyper-enhancement of epicardial fat along the course of the thickened main coronary artery walls. One month later, the recipient underwent re-transplantation due to progressive worsening of the clinical condition and refractoriness to intravenous medication. The anatomopathological findings of the explanted heart provided impressive visualization of structural and histopathological changes. These results could guide the tailoring of preventive therapeutic strategies and non-invasive monitoring of cardiac grafts.

## Introduction

Cardiac transplantation remains the most effective treatment for end-stage heart failure with excellent short- and long-term survival rates (1). Cardiac transplant recipients undergo extensive postoperative screening to exclude post-transplant complications. Chronic sequelae, such as cardiac allograft vasculopathy, play an important role in the post-heart transplantation course (2). Cardiac magnetic resonance imaging (CMRI) is a non-invasive, non-irradiating diagnostic tool used to monitor graft health and estimate the onset of tissue rejection processes or myocardial fibrosis (3). In this study, we present a case of a young heart transplant recipient undergoing re-transplantation due to severe accelerated cardiac allograft vasculopathy (CAV). We also report the striking similarities between the CMRI results and histopathological samples obtained from the explanted heart.

## Case description

**Figure 1:**
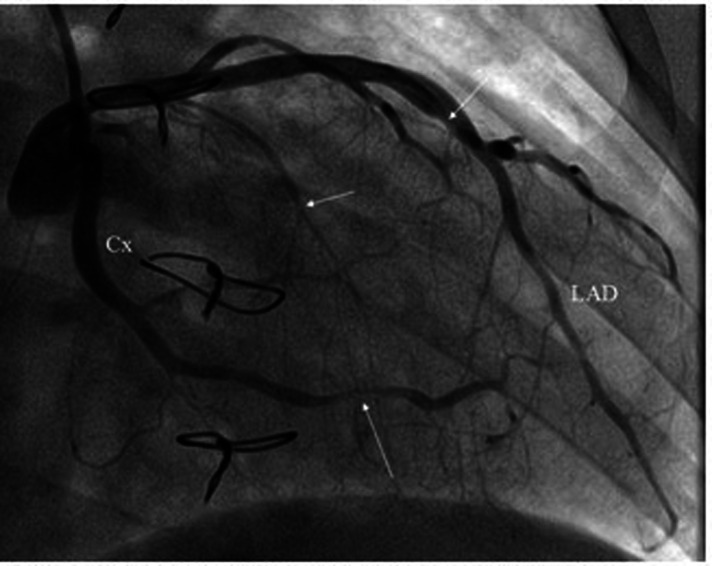
Invasive coronary angiography. CX, circumflex coronary artery; LAD, left anterior descending coronary artery.

**Figure 2:**
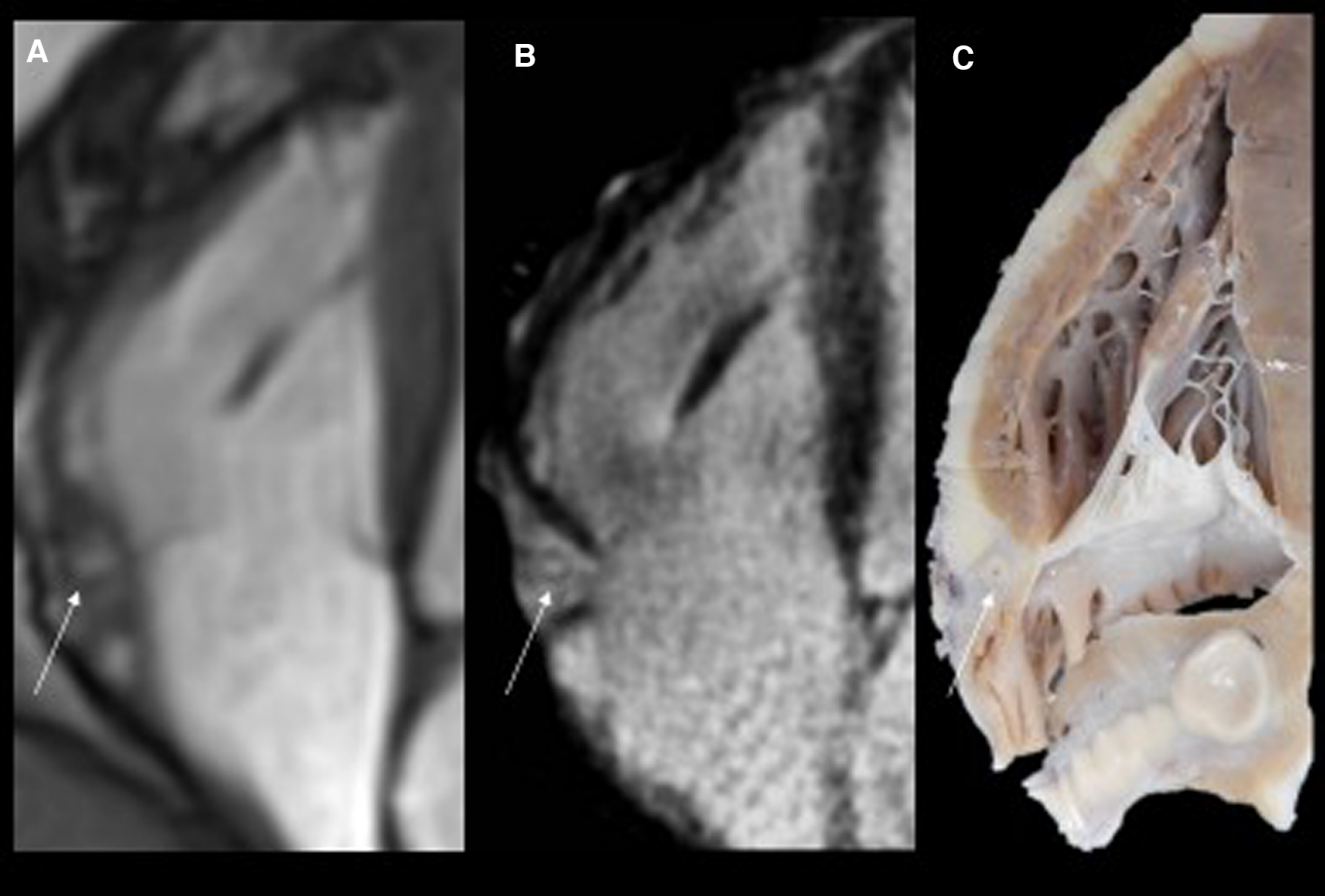
Routine magnetic resonance (MR) cine images in the four-chamber view (panel **A**) show thickened and hyperintense vessels (arrow) at the right atrioventricular groove. The high resolution T2-weighted and fat saturated sequence (panel **B**) at the same level confirms thickened walls of the right coronary artery with hyperintense epicardial fat tissue due to edema. The corresponding specimen slice confirmed significant epicardial coronary vessel wall thickening, particularly in the right coronary artery (RCA, arrow).

**Figure 3:**
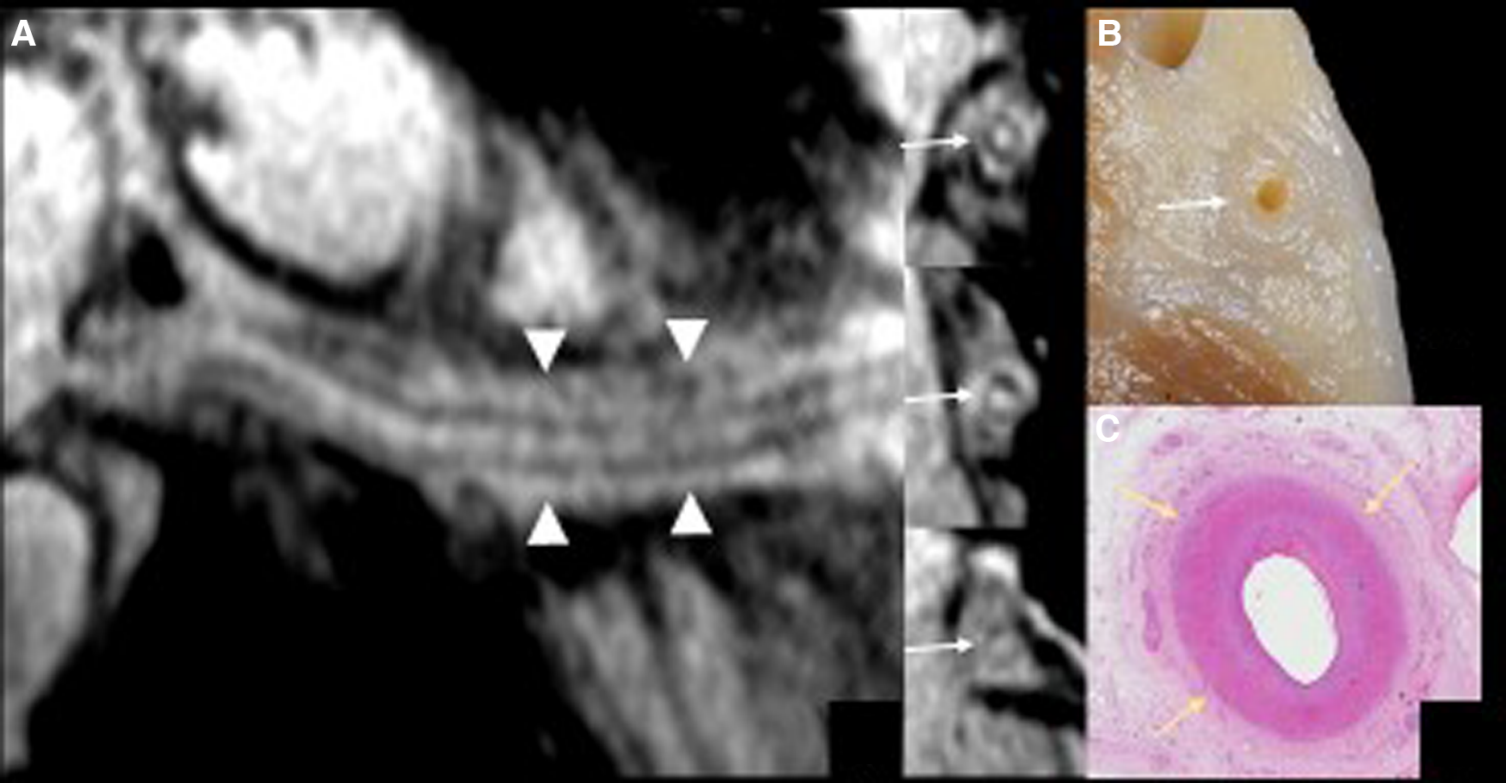
The curved multiplanar reformatting view (panel **A**) of the right coronary artery with the 3 short-axis vessel views using high resolution magnetic resonance (MR) coronary imaging with additional fat epicardial suppression (whole-heart imaging) confirms wall thickening along the course of the right coronary artery (arrows) and inflammatory abnormalities of the epicardial fat surrounding the vessel (arrowheads). The detailed anatomical view of the right atrioventricular groove (panel **B**) matches the MR coronary findings.

A 29-year-old female heart transplant recipient presented to our care center with a generalized poor health condition. The patient was affected by congenitally corrected transposition of the great arteries with a subpulmonary interventricular defect and right ventricular hypoplasia. In 1998 at the age of twelve the patient underwent Glenn atrial switch procedure. In September 2015, heart transplantation was performed for severe heart failure refractory to therapy and episodes of paroxysmal supraventricular tachycardia that required frequent hospitalization. Due to residual claudication caused by severely impaired lower limb muscles, the patient was unable to undergo a stress test during periodical follow-up or during hospitalization. Upon admission to our care center, the patient appeared pale and complained of dizziness and fatigue. The patient's blood pressure was 123/70 mmHg with a heart rate of 105 beats/min. The 12-lead electrocardiogram (ECG) showed sinus tachycardia, first degree atrioventricular block, right bundle branch block, and diffuse non-specific abnormalities of ventricular repolarization. Transthoracic echocardiography revealed a slightly reduced left ventricular ejection fraction (46%). Laboratory findings showed a normal cardiac troponin I level (5.4 pg/ml) and an elevated terminal pro brain type natriuretic peptide level (353 pg/ml). Invasive coronary angiography showed severe stenosis at the distal portion of the circumflex coronary artery and diffuse sclerosis of the left anterior descending coronary artery. Intravascular ultrasonography showed a Stanford scale IV score (Figure 1). Consequently, the therapeutic strategy was revised and low-dose bisoprolol, ace inhibitors, and diuretics were added to the remaining medication, which comprised cyclosporine, everolimus, pravastatin, and aspirin. At day 7 of hospitalization echocardiography showed improvement of the left ventricular function (58%) and a reduction of the N-terminal pro brain type natriuretic peptide level (235 pg/ml). The symptoms disappeared and the patient was discharged with a scheduled CMRI 7 days later. CMRI was performed using a 1.5 Tesla scanner (Magnetom Aera 1.5 T; Siemens, Erlangen, Germany) and showed mild concentric hypertrophy of the left ventricle and moderately reduced left ventricular function. T2-weighted short-tau inversion recovery (STIR) sequence images showed widespread sub-epicardial hyperintensity of the myocardial segments (signal intensity ratio to skeletal muscle > 2) along the course of the coronary arteries (Figure 2). T2 mapping sequences showed an elevated value of 57 ms, and the myocardial native T1 values and extracellular volume percentage were significantly elevated (1170 ms and 41%, respectively). Late gadolinium enhancement (0.2 mmol/kg Gadoteric Acid, Dotarem®, Guerbet, France) demonstrated diffuse sub-epicardial hypersignal along the lateral, free, and left ventricular walls. The “whole-heart” sequence (navigator-gated, vector ECG-triggered, fat-suppressed T2-weighted 3-dimensional gradient-echo inversion recovery) demonstrated widespread hyper-enhancement of epicardial fat along the course of the thickened main coronary artery walls (Figure 3) At rest, there were no sub-endocardial defects in the anteroseptal and basal infero-septal segments. Fifteen days after the CMRI was performed, the patient experienced an episode of lipothymia at home and was re-admitted through the emergency department. Due to the rapid and progressive worsening of the patient's clinical condition, which included severe hypotension and the need of inotropic drugs, the patient was placed on the transplant waiting list and underwent re-transplantation in May 2017. At the last follow-up, the patient was in a good condition and will be continuously followed at our dedicated heart transplant clinic.

## Discussion

**Figure 4:**
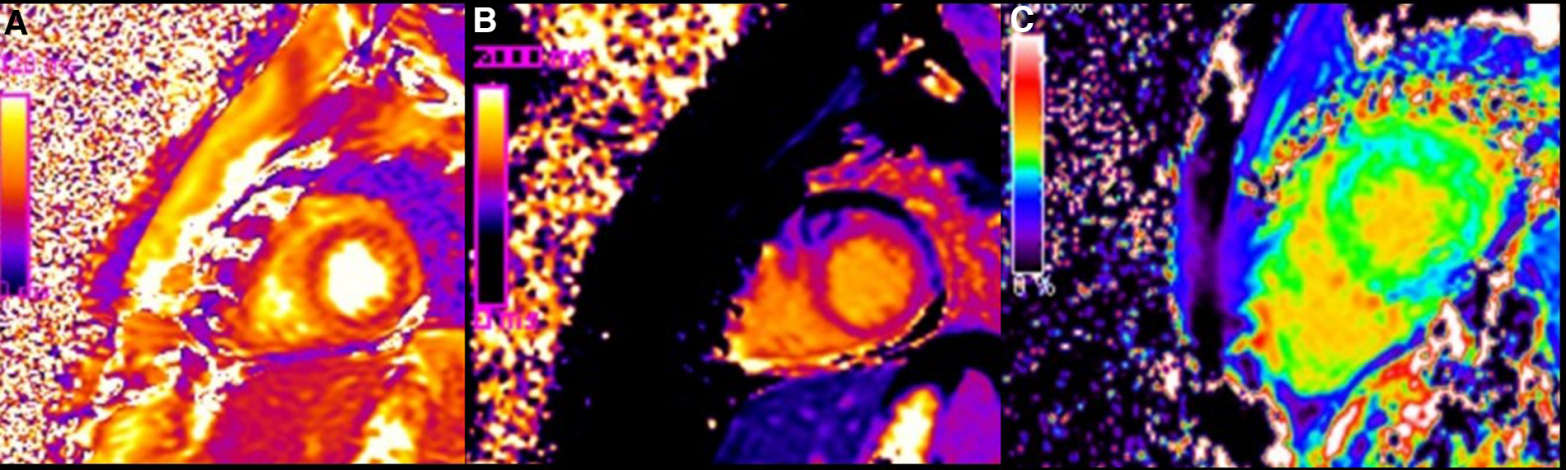
Color coded images of T2 map (**A**), native T1 map (**B**) and ECV map (**C**), demonstrating diffuse increase of T1 and T2 relaxation (on quantitative analysis native T1 was 1200 ms and T2 was 60 ms) and elevation of extracellular volume fraction (ECV: 30%) present in condition of diffuse oedema and diffuse fibrosis.

**Figure 5:**
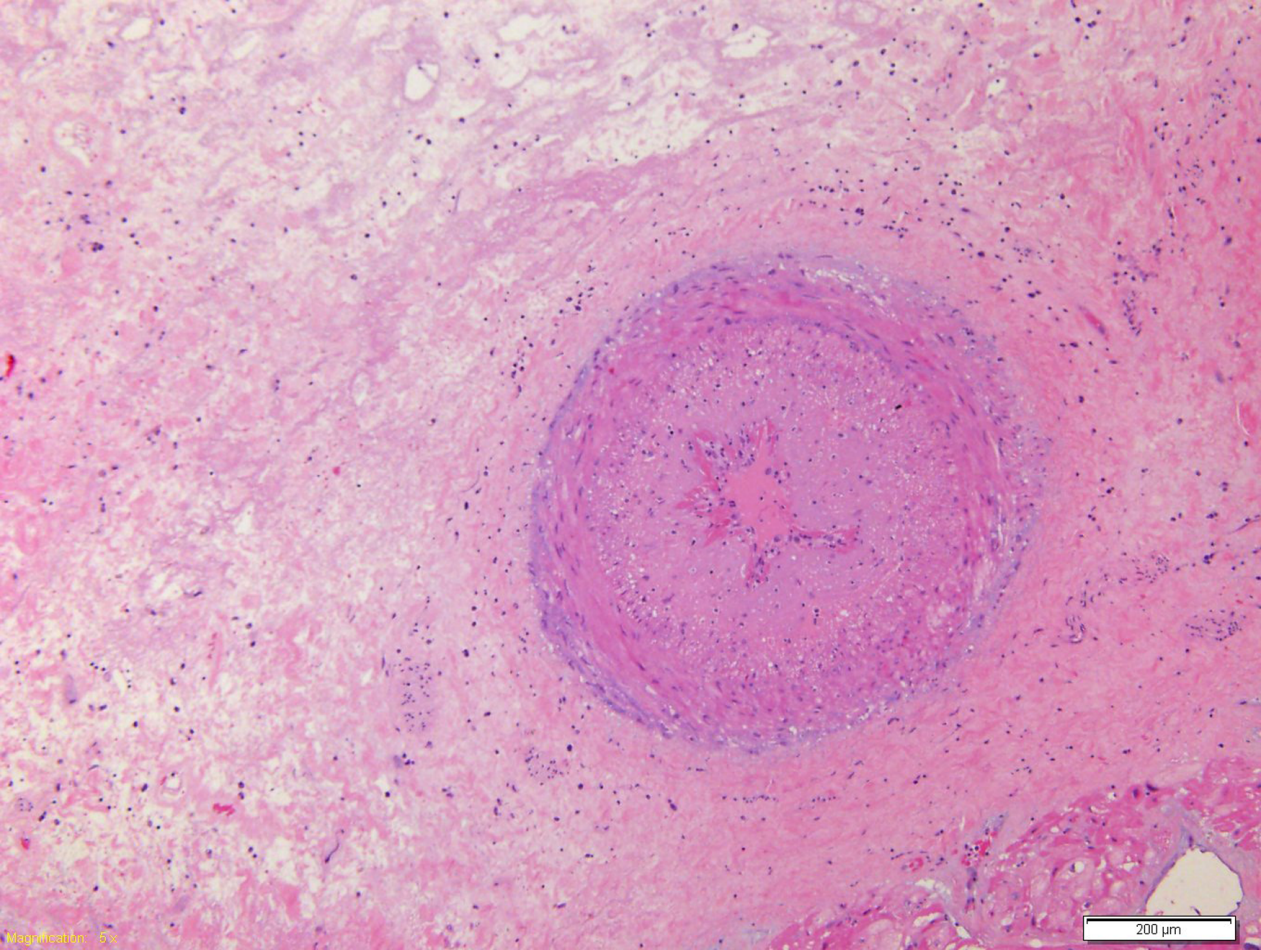
Coronary branch characterized by marked hyperplasia of the intima which causes almost complete obliteration of the lumen.

**Figure 6:**
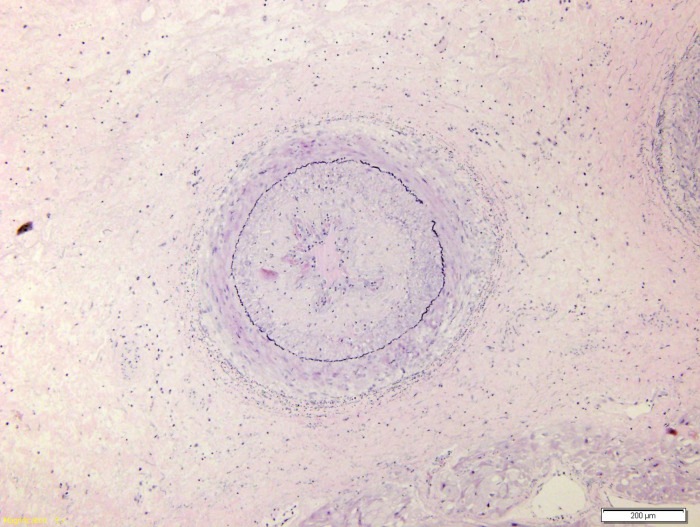
Trichrome-EVG show an intact internal elastic lamina only focally fragmented (EVG, 10x).

CMR imaging-based myocardial tissue characterization with T1 and T2 mapping has emerged as a non-invasive and highly sensitive method of detecting cardiac allograft rejection, with numerous studies demonstrating good correlation between CMR-based mapping and histopathology-determined rejection (1). A single CMRI study can provide information on cardiac volumes, function, wall motion, tissue characterization, and ischemia at rest, pharmacological stress testing, and myocardial perfusion reserve assessment, which is an important prognostic factor in the evaluation of heart transplant recipients. CMRI can also identify edema, which is often associated with acute heart transplant rejection or myocarditis. Strategies to detect myocardial edema include semi-quantitative measures, such as T2 signal intensity ratios, or quantitative techniques, such as T2 mapping (2, 3). CMR imaging-based myocardial tissue characterization with late enhancement sequences (4) and sequences such as T1 and T2 mapping has emerged as a non-invasive and highly sensitive method of detecting cardiac allograft rejection. Numerous studies demonstrated good correlation between CMR-based mapping and histopathology-determined rejection (1). In our case, CMRI showed non-specific elevation of inflammatory indices, increased T2 mapping values (Figure 4), and widespread sub-epicardial hyperintensity along the course of the coronary arteries on STIR sequences (Figure 3). We observed an impressive correlation between CMRI, invasive coronary angiography images, and increased endocardial fibrosis of the circumflex coronary and left anterior descending arteries in the explanted heart (5). Interstitial inflammatory cellularity along the course of the coronary arteries correlated with the CMRI images; however, it was not detectable on the invasive coronary angiography images (Figure 1). Some authors have described the importance of the myocardial perfusion reserve (MPR) index as the only parameter able to independently predict microvascular and macrovascular CAV (3, 6). In our young girl recipient, it was not possible to estimate MPR because of her worsening clinical condition. However, first-pass perfusion imaging performed on the patient at rest did not demonstrate subendocardial defects. At late enhancement sequences, our patient did not show the typical pattern of late gadolinium enhancement, usually related to severe CAV on angiography, as some studies described (3, 7). However, we found an unusual extensive hyperenhancement of epicardial fat along the course of the main coronary arteries and diffuse subepicardial enhancement with a corresponding diffuse edema (Figure 3). In addition, myocardial T1 mapping showed elevation of native T1 mapping and ECV (Figure 4), providing a quantitative measurement of extracellular volume expansion such as a more sensitive marker of adverse change within the cardiac allograft. Examination of explanted heart showed distal branches of main subepicardial coronary arteries and small peripheral vessels characterized by severe intimal proliferation, sometime till complete obliteration of the lumen (Figure 5) without necrosis, calcification and cholesterol cleft, with an intact internal elastic lamina (Figure 6) these findings are consistent to the CAV demonstrated by coronary angiography. No myocardial infarction or diffuse interstitial scarring was evident, particularly along the coronary arteries, owing to perivascular fibrous tissue being poorly vascularized and focally infiltrated by sparse mononuclear cells. These findings could be related to the development of acute perivascular inflammation, which was probably immune-mediated before intimal proliferation and, therefore, before the sequelae of myocardial scarring occurred. Existing methods for monitoring the allograft are invasive and may be insufficiently sensitive. Advanced methods, such as CMRI, may be routinely utilized to evaluate cardiac allograft vasculopathy progression and help to define the best immunosuppressive protocol strategy.

## Conclusion

To our knowledge, this is the first report on the CMRI findings in allograft failure and its relation to histopathological specimens. This concept could raise the possibility of tailoring preventative therapies.

## Data Availability

The raw data supporting the conclusions of this article will be made available by the authors, without undue reservation.
